# Supplementary material for: Highly Sensitive and Selective Detection of L-Tryptophan by ECL Using Boron-Doped Diamond Electrodes
Source: Sensors (Basel). 2024 Jun 4;24(11):3627. doi: 10.3390/s24113627 (PMC11175342; doi:10.3390/s24113627)
Supplement: Supplementary file 1 [file sensors-24-03627-s001.zip › sensors-3036273-supplementary.pdf]

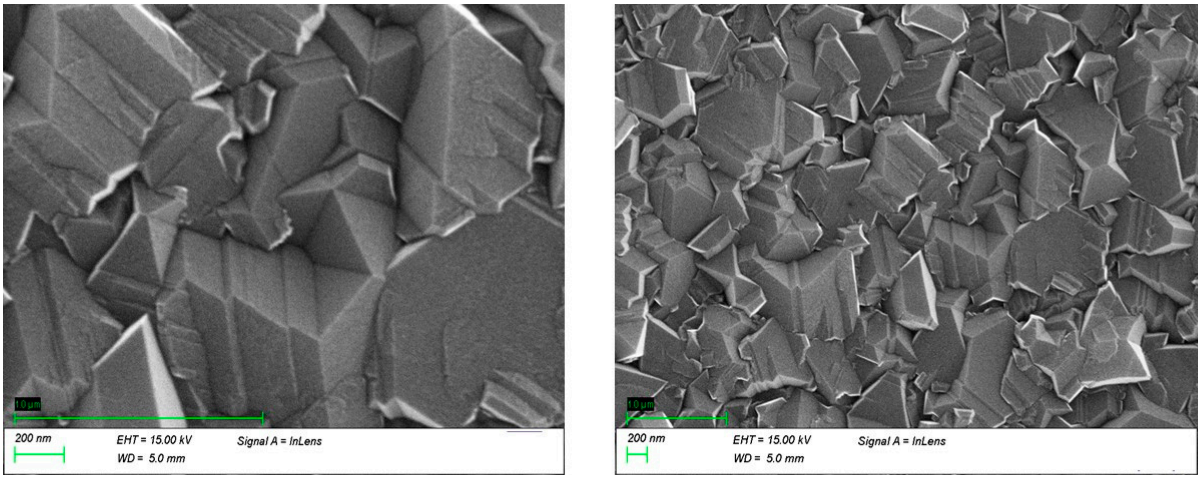

Figure S1: Typical scanning electron microscope SEM images of the surface of the BDD electrodes (worker or counter electrodes)

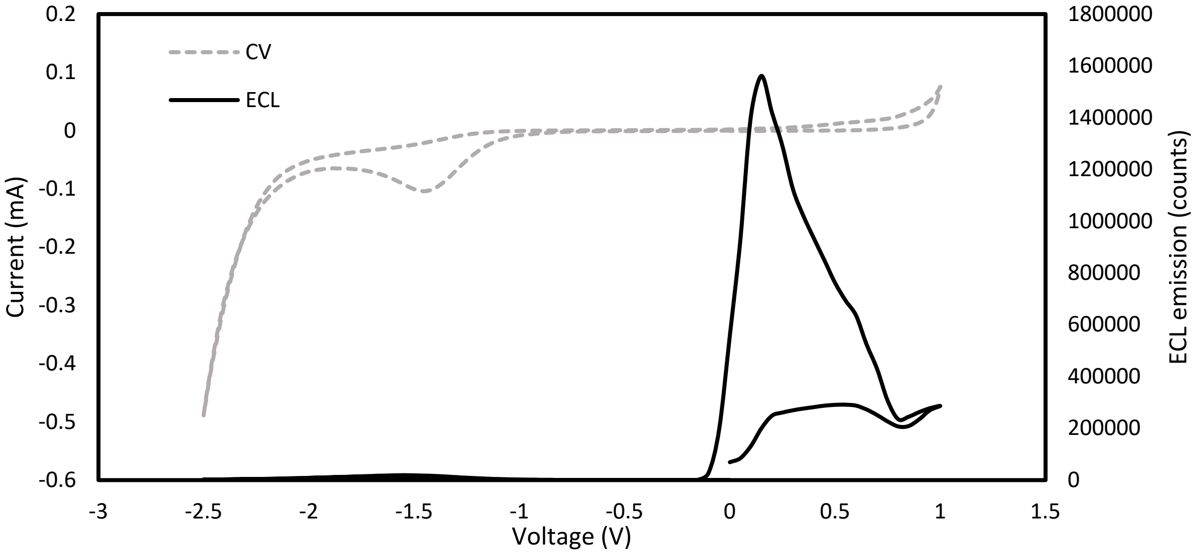

Figure S2: Typical response of BDD electrode to a 0.1 M PBS solution containing 1 µM luminol. CV scan was 0 V → - 2.5 V → 0 V → 1 V, at a scan rate 0.1 V/s. CV (grey dotted line) ECL emission (black line)

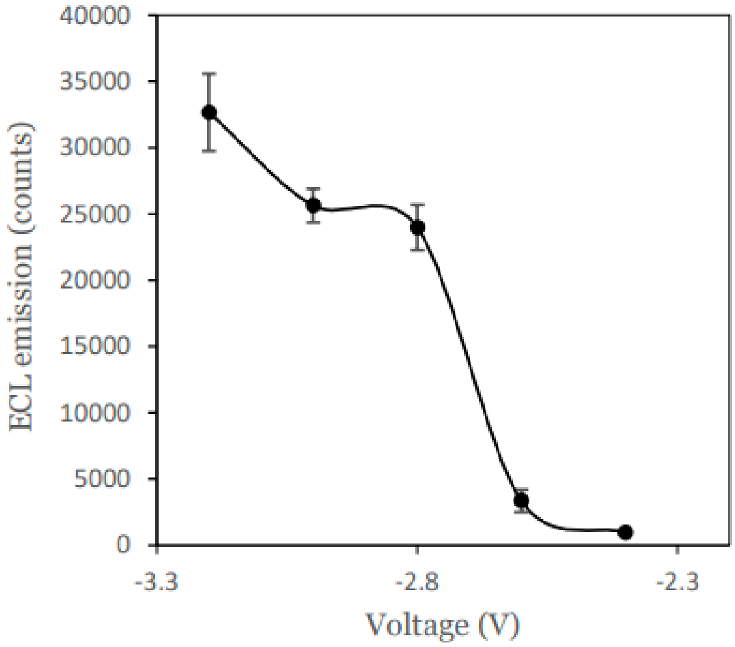

Figure S3: Influence of the lower cathodic potential on the ECL emission of 500 nM of L-Tryptophan in 0.1 M PBS. Scan 0 V ⟶ x V ⟶ 1.5 V ⟶ 0 V at a scan rate of 0.1 $V.s^{-1}$

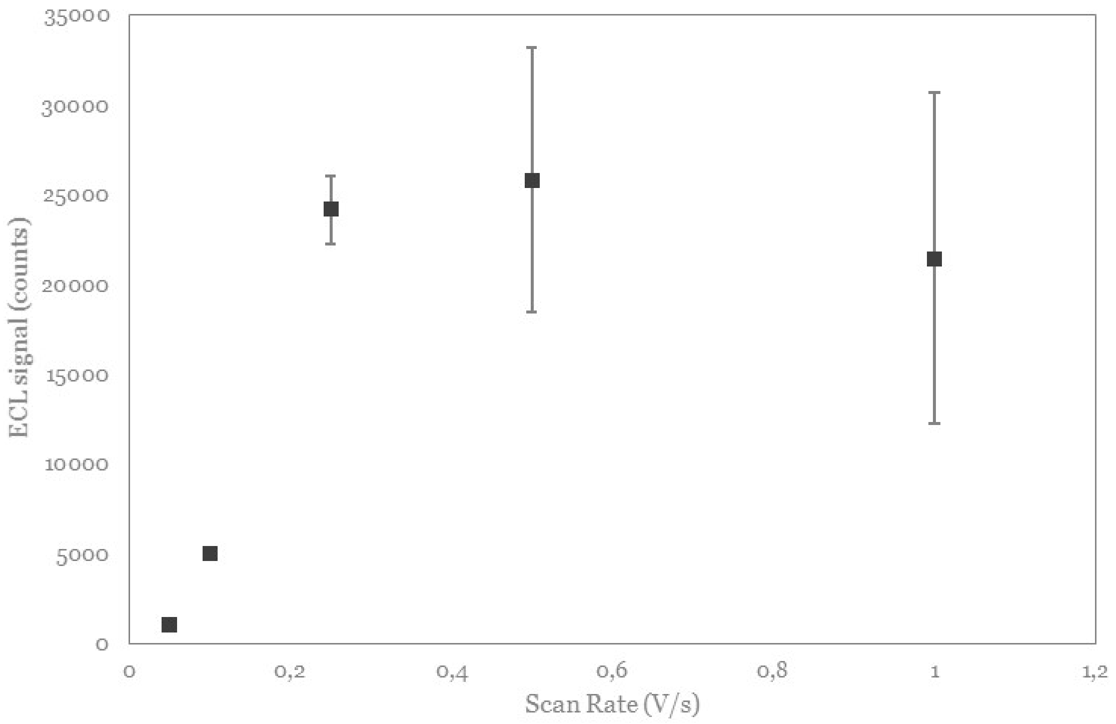

Figure S4: Influence of scan rate on the ECL emission of 500 nM of L-Tryptophan in 0.1 M PBS. Scan 0 V ⟶ -3V ⟶ 1.5 V ⟶ 0 V.

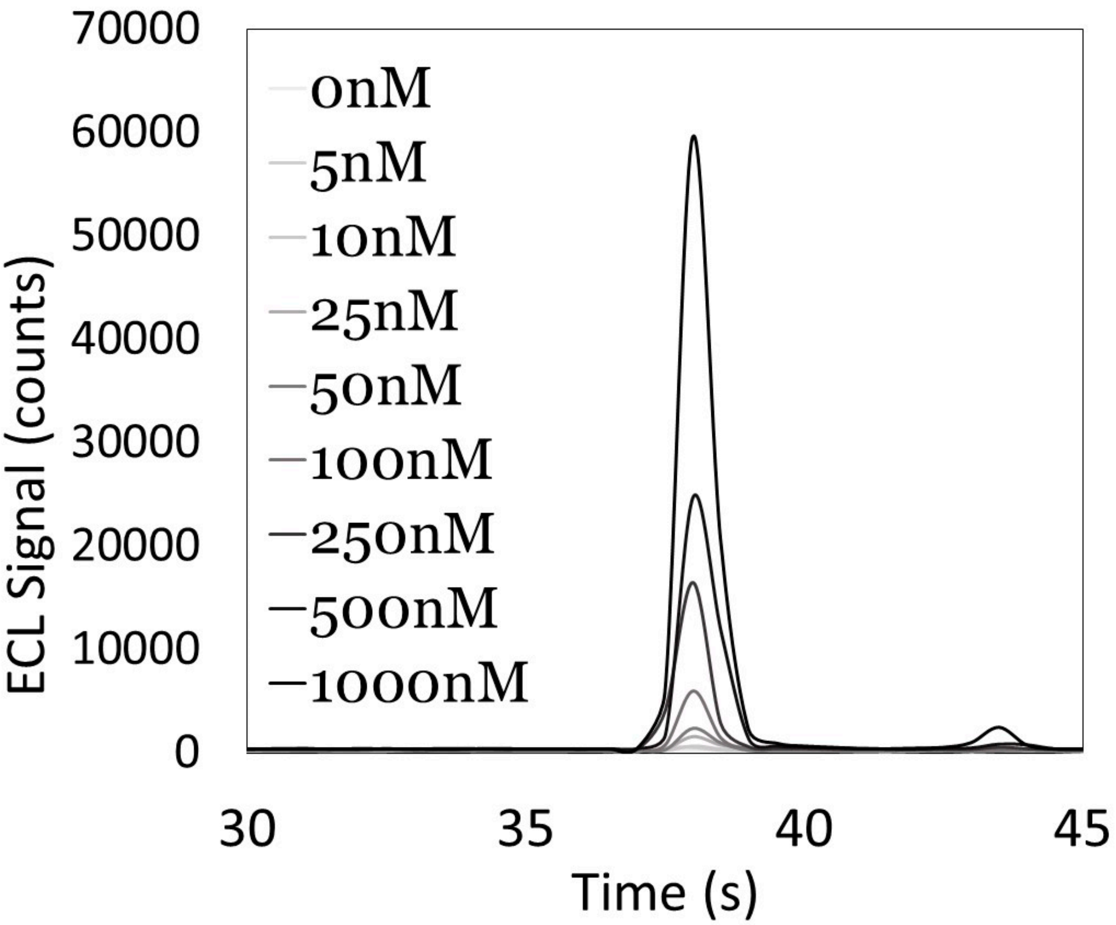

Figure S5: ECL peak emission for each concentration of tryptophan measured (0, 5, 10, 25, 50, 100, 250, 500, 1000 nM) in 0.1 M PBS (pH 7.4) on BDD electrodes after successive reduction and oxidation
